# A Multicomponent Preventive Intervention in the Early Elementary Years: A Look at Academic and Social Adjustment Outcomes

**DOI:** 10.1007/s11121-024-01748-w

**Published:** 2024-11-11

**Authors:** Ronald J. Prinz, Emilie P. Smith, Brianna Tennie

**Affiliations:** 1 Research Center for Child Well-Being, University of South Carolina, Columbia, SC USA; 2 Department of Human Development and Family Studies, Michigan State University, East Lansing, MI USA

**Keywords:** Multicomponent prevention, Child conduct problems, Academic enhancement

## Abstract

Cogent indicated prevention with young children at risk for early onset conduct problems needs to address multiple domains of influence in school and home settings. A multicontextual preventive intervention (MPI) spanning grades one and two was conducted in schools serving economically disadvantaged communities and evaluated separately for boys and girls. The cluster randomized design evaluated children nested within schools receiving either the MPI (6 schools), which consisted of after-school reading-mentoring, home-based family, peer coping-skills, and classroom components, or a control condition (6 schools) involving a school-wide conflict management program without targeted intervention. Drawn at the end of kindergarten based on elevated behavioral difficulties and first-grade attendance at one of the 12 schools, the two subsamples consisted of 193 boys and 171 girls (63% in households with annual income < $15,000; 95% Black children). Extensive fidelity data indicated that the MPI components were well implemented. The two post-intervention third-grade outcomes in this report are academic performance and social/behavioral adjustment. The analyses involved a linear mixed effects model controlling for school. The key finding for the male subsample was that the MPI produced greater overall and language-arts/reading achievement, measured by report cards for the entire third-grade school year, compared with the control group. MPI-control differences did not emerge for externalizing problems and social competence assessed via teacher and parent report. In the face of elevated risk and poverty, the study underscored the importance of contributions from community-based reading-mentors, positive and inclusive classrooms, and nurturing family contexts in achieving academic gains.

**Supplementary Information:**

The online version contains supplementary material available at 10.1007/s11121-024-01748-w.

## Introduction

Multicomponent preventive interventions for children are undertaken with the general assumption that multiple settings and domains can exert collective influence on the targeted outcomes. Prevention of childhood early-onset conduct problems is no exception. Behavioral difficulties in young children present elevated risk for academic struggles, mental health problems, strained relationships with peers and adults, and adolescent substance use (Bierman & Slotkin, 2023; Galán et al., 2020; Prinz, 1998). Indicated prevention efforts in this area have recognized the importance of parenting, classroom and teacher impact, and interactional skills with peers, to impact not just behavioral adjustment but also academic development (Bierman et al., 2020; Cavaleri et al., 2011; Henry et al., 2012; McMahon & Pasalich, 2020; Pas et al., 2015; Smith et al., 2004; Weare & Nind, 2011; Wilson & Lipsey, 2007). Eco-developmental models recognize that these various contexts emerge in increasing levels of importance as children grow and expand their social interactions (Hawkins et al., 2007; Tolan et al., 2003). These models offer strengths-based perspectives that highlight the role of supportive family and school relationships meant to foster more positive behavioral and academic outcomes (Bierman et al., 2020; Dishion & Patterson, 2015; Henry et al., 2012).

For young children already exhibiting behavior problems, academic risk when left unaddressed has the potential to escalate into cascading effects. When poverty is a co-occurring contextual factor, the timely strengthening of academic skills through a concerted prevention strategy becomes an even greater but challenging imperative (Bierman & Slotkin, 2023; Dishion & Patterson, 2015; Hinshaw, 1992). This issue is particularly germane for Black children given reported achievement gaps by race and gender, albeit the racial gap though persistent across social class has been decreasing over the past few decades (de Brey et al., 2019). Although Black girls and boys perform at similar levels in early elementary school, after second grade the differences grow so that by middle school the achievement of girls exceeds that of boys (Davis, 2003; McMillian et al., 2011; Mickelson & Greene, 2006), underscoring the need to examine intervention impact separately by gender. Enhancing the achievement of Black males could be pivotal, especially given that achievement and social development are so closely tied. Black boys and girls both experience disproportionate discipline for similar infractions than other children (Gregory & Fergus, 2017). Leveraging prevention science in creating “promotive” family, school, and community contexts that create an environment of warmth, belonging, and engagement for minoritized students is a promising pathway towards fostering achievement and well-being (Bradshaw et al., 2021; Coll et al., 1996; Durlak et al., 2010; Smith et al., 2016).

This paper focuses on a multicomponent intervention spanning first and second grades that was tested in a cluster-randomized controlled intervention study undertaken in underserved communities with a predominantly Black population of children, evaluated separately by gender. The four-component multicontextual preventive intervention, which included reading-mentoring, family, peer coping-skills, and classroom program components, was grounded in a social-interactional coping-competence model organized around risk and protective factors that contribute to the development of early-onset conduct problems and co-occurring academic difficulties (Blechman et al., 1995; Prinz et al., 2000). Four conceptual themes ran through all the intervention components: (1) enhancement of prosocial coping strategies through repeated opportunities for practice in different contexts, (2) regular recognition for attainable success, (3) building of positive relationships with other children and significant adults, and (4) development of regular, positive communication between school and home (Blechman et al., 1995; Dumas et al., 1999a, b; Prinz et al., 2000). These skills map onto important aspects of positive youth development including competency, confidence, and connection (Lerner et al., 2021). Together, these themes reflect the assumption that children are most likely to use the skills they develop through each intervention component if practice enables them to feel competent at what they do and if they become convinced that these skills have a positive impact on how others respond to them in different settings.

This article was written for the *Prevention Science* special issue commissioned to recognize the scientific contributions of Dr. Robert J McMahon and honoring his retirement. Among his many accomplishments, Dr. McMahon was one of the founding PIs of Fast Track, a landmark investigation of a multicomponent preventive intervention addressing risk for early onset conduct disorder in children (Bierman et al., 2020; McMahon et al., 1999). With some similarities to the present study, the Fast Track cluster-randomized trial implemented a multicomponent intervention starting in first grade with boys and girls and then sustained the intervention through second grade, notwithstanding that Fast Track’s intervention was extended well beyond second grade.

Childhood conduct problems are subject to gender variation with respect to base rates and developmental trajectories (Gutman et al., 2018; Meier et al., 2011; Zahn-Waxler et al., 2008) and potentially with respect to the impact of preventive interventions (Beelmann & Lösel, 2021; Conduct Problems Prevention Research Group, 2010; Webster-Stratton, 1996). Accordingly, it was important in the present study to evaluate intervention impact on boys and girls separately to pinpoint any differential gender effects that might emerge.

The present study involved the implementation of the multicomponent intervention in a cluster randomized trial with young children at elevated risk for early onset conduct disorder and associated academic difficulties. The goals of this article are as follows: (1) to elucidate the nature, conceptualization, and rationales for each intervention component in the context of the efficacy trial; (2) to convey the results of meticulous assessments regarding intervention fidelity; and (3) to convey the results for the two primary third-grade outcomes, academic performance, and social/behavioral adjustment, for boys and girls separately.

## Method

### Overview and Design

This study focused on the implementation and evaluation of a multicontextual preventive intervention (MPI) extending across first and second grade applied to male and female subsamples of young children showing elevated behavioral and academic risk. Conducted in a medium-sized southeastern community during the period 1998–2004, the design included 12 elementary schools that served economically disadvantaged neighborhoods. The 12 schools were stratified based on school size and relative socioeconomic level and then randomly assigned to either the MPI, with indicated components administered to children and families coupled with classroom-wide programming, or to the control condition which involved a school-wide conflict management program with no programing administered to individual children or families. Employing this type of control condition allowed these schools to receive potentially beneficial programming without the implementation of programming for individual children, boosting the schools’ interest in participating. The design involved three yearly consecutive cohorts at all 12 schools (Prinz et al., 2000).

### Study Subsamples

The subsamples for this study were created through a multiple-gating procedure involving screening of children on the Aggression Scale of the Child Behavior Checklist-Teacher Report Form (CBCL-TRF; Achenbach & Rescorla, 2001) by kindergarten teachers and on the CBCL (Achenbach, 1999) by parents/caregivers. Given gender specific norms for the CBCL-TRF and CBCL Aggression Scale, separate male and female subsamples were formed. Within each gender group, inclusion in the respective subsample required: (1) scoring in the top 20% of kindergarten children on the Aggression Scale for both the CBCL-TRF and the CBCL or scoring in the top 10% of kindergarten children on the Aggression Scale for the CBCL-TRF only; (2) parental/caregiver consent; and first-grade attendance at one of the twelve participating schools. The study’s CONSORT diagram is found in Fig. 1, online.

The subsample of boys consisted of 104 and 89 children in the MPI and control groups respectively, and the subsample of girls included 100 and 71 children in the MPI and control groups. The initial demographic characteristics for the subsamples as a function of study group are summarized in Table 1, online. Within each of the two subsamples, the MPI and control groups were comparable with respect to demographic characteristics. For all participants overall, approximately 95% of the children were Black and 5% White; for parent/caregiver educational attainment, 60% achieved high school graduation or less; and 63% of the children resided in households with less than $15,000 in annual household income (and 85% less than $30,000).

By the end of third grade across the three cohorts and both subsamples, the research team was able to retain 92.97% of the children in the study.

### Preventive Intervention Components

The MPI consisted of four components, which are described in substantial detail in Dumas et al., (1999a, b) and are summarized below. The reading-mentoring, family, and peer components were targeted intervention strategies, while the classroom component was universally administered to whole classes of children.

#### Reading-Mentoring Program Component

The reading-mentoring program (RMP) involved reading tutorial and enrichment in a group context involving supervised mentors and fun activities. The RMP was designed to foster language and early reading skills, and more generally promote early academic achievement. The conceptual underpinnings for the RMP are multiple: (1) language/communication and reading skills go hand in hand, especially for children having behavioral and social difficulties (Blechman et al., 1995; Chow & Wehby, 2018; Warr-Leeper et al., 1994); (2) young children with better developed language and early reading skills are better able to navigate academic activities and less likely to spend time off-task or engaging in problem behavior; (3) acquisition of reading skills depends on attentional and social-behavioral variables and is critical over time to achievement in other academic areas (Fergusson & Horwood, 1995; Hinshaw, 1992; Wentzel, 1993); and (4) reading-mentoring is designed to help children develop a lifelong interest in reading and an identity as a learner (Werner, 1995). The tutorial component, a central part of the RMP, was based on the manualized and empirically validated approach developed by Juel and colleagues and emphasized academically appropriate reading, word study, and phonetics (Johnston et al., 1998; Juel, 1996; Juel & Minden-Cupp, 2000). The reading activities were tailored to meet each child’s level. Each 45-min tutorial session was comprised by three tasks: (1) reading familiar material on repeated occasions to encourage the child to practice reading to the reading-mentor; (2) word study in which creative games and tasks were used to teach phonetic knowledge, expand vocabulary, and incorporate writing tasks; and (3) introduction of new material through which the reading-mentor modeled by reading to the child. The reading materials were selected from available grade-level appropriate materials, representing the background and experiences of the participants, coupled with training of the reading-mentors communication and relational skills congruent with culturally relevant pedagogies (Ladson-Billings, 1995). The RMP also included group enrichment and enjoyable activities, snacks, and reinforcement of cooperative behaviors using tokens exchangeable for group bonus activities. High school and college students who served as reading-mentors received programmatic training and onsite supervision. The RMP was conducted for 3 ½ hours after school twice a week on most weeks, throughout the first and second grades, and varied in total from 73 to 88 tutorial sessions across the six MPI schools. Children’s median attendance at RMP tutorial sessions was 81%.

#### Family Intervention Program Component

Every child’s parent (or primary caregiver such as a custodial grandparent) received a home-based family intervention program (FIP). The FIP was predicated on recognition of the critical role that parents and other primary caregivers play as socializing agents who affect child behavior at home and beyond. The program’s foundation derives from a social-interactional and social learning framework for parenting and family support (McMahon & Pasalich, 2020; Miller & Prinz, 1990; Patterson et al., 2010; Prinz & Dumas, 2004; Serketich & Dumas, 1996). Much of the FIP involved parenting skill-building to foster prosocial and academic child development. Interspliced throughout scheduled sessions, and occasionally in crisis situations, the family consultants provided parent/caregiver support to address personal and family concerns. The personal support facet, developed and tested in prior work, was shown to enhance parental engagement during intervention (Miller & Prinz, 2003; Prinz & Miller, 1994). The FIP was delivered by professional family consultants, employed full-time by the project, who were recruited from participating communities and extensively trained on all aspects of the protocol during five full-time months involving didactic sessions, extensive roleplay, and practice sessions with volunteer families. Programing extended over first and second grades (including summers), with the home-visit sessions scheduled more frequently during the first 12 months and then spaced out after that. The ten modules for FIP included: reinforcing Good News Notes (from school-home communication); pinpointing child behaviors; attending and ignoring; praise and criticism; communicating with school officials; consequences for changing unwanted behaviors; when adults make requests; revisiting positive parenting practices; working on caregiver goals for their child; and coaching prosocial interactions. The support element, which was integrated into every home-visit session, involved prompting for parent concerns and applying facilitative listening and problem-solving without imposing solutions. The advocacy element dealt with a broad array of needs related, for example, to school issues, childcare, social services, healthcare, legal aid, and work setting. The median number of home sessions for families was 14, with 80% of the families participating in 7 or more home sessions; 10% of the families participating in fewer than 4 home sessions, while 10% participating in greater than 26 home sessions.

#### Peer Intervention Component

The peer intervention component, called Peer Coping Skills (PCS), targeted communication skills and self-control with other children through a small-group program delivered by trained staff. The timing and content of PCS are predicated on the observation that children who exhibit high rates of aggressive behavior when they enter first grade are at risk for experiencing pronounced peer rejection and developing a negative reputation that is very difficult to undo later. PCS group sessions involved many brief opportunities to practice information exchange and other prosocial communication skills in a series of probes and interaction tasks, accomplished with modeling, coaching, and positive feedback. There was no lecturing or didactic content in this program. In a prior randomized trial, PCS increased observed prosocial communication skills and reduced teacher-reported aggressive behavior in a diverse sample of young children (Prinz et al., 1994). In the current study, PCS was conducted throughout first grade, with the intention of teaching behaviorally at-risk children in small groups (*n* = 8) that also included equal numbers of socially competent children, to communicate effectively with peers and potentially reduce their risk of developing disturbed peer relations. The program relied on group discussions, structured group activities, and communication role plays designed to impart effective communication skills through specific practice opportunities. Each PCS group met for 20–22 sessions, each 50 min in duration. The median number of PCS sessions attended was 17, with 90% of the children attending 10 or more sessions.

#### Classroom Program Component

The overall thrust of the classroom program, which was implemented in all first and second grade classes in the intervention schools, was to promote a positive climate, empower teachers with effective classroom management strategies, and provide frequent recognition for prosocial and other constructive child behaviors. A key part of the classroom program, the Good Communication Game, is a teacher-administered classroom management program for enhancing group functioning and expanding prosocial coping skills, and a variant of the Good Behavior Game (Barrish et al., 1969; Blechman et al., 1981). Also included was the Good News Note system (GNN), which provided frequent and positive communications between school and home regarding discrete behavioral and academic successes by individual children. The GNN is a variation of what has also been referred to as the “daily behavior report card” or “school-home communication” strategy (Vannest et al., 2010). However, with the GNN teachers only convey positive child behaviors and academic successes so that reinforcement was bolstered, and the system did not become aversive to the child. The classroom program also started with a teacher-training workshop on effective communication, classroom management, and program-element administration, and was sustained through recurring supportive consultation with the teachers.

#### Delivery Agents for the Intervention Components

The delivery agents for the family, peer, and classroom components, all from the local community and matching the racial makeup of child and family participants, were professionals employed full-time on the project who received in-service training for their respective intervention protocol. Each family consultant had a masters’ degree in a counseling related field, and staff conducting the PCS and classroom programing were experienced teachers. For the RMP, the male and female high school and college students deployed as reading mentors included substantial Black representation.

#### Procedures for Assessment and Documentation of Intervention Fidelity

Substantial effort for each of the three targeted intervention components went into the tracking, promotion, and documentation of process and content fidelity. Process fidelity refers to the manner with which the intervention was supposed to be delivered, while content fidelity denotes the core actions and activities expected when the intervention’s integrity is maintained. Process and content fidelity of the RMP was evaluated across children, reading mentors (tutors), time of year, schools, and cohorts. A total of 345 randomly sampled reading-mentoring sessions were observed in vivo and rated by independent fidelity raters. The RMP process dimensions included attentive listening; showing encouragement, respect, and concern; giving short and clear instructions; dealing effectively with disruptions or challenges; appearing organized and well-prepared for the tutoring session; and using praise and gentle correction appropriately. The RMP process categories included adequate written plan for tutoring session, starting session by reading from a book, word-study activity emphasizing phonics and fluency, timed reading for fluency (early readers) or reading from a new book (emergent readers), asking comprehension questions, and writing exercise linked to reading activity. With respect to the FIP, 20% of audiotapes for home-delivered family sessions randomly selected across family consultants, families, cohorts, and time periods were systematically evaluated at a remote site with respect to process and content fidelity. Based on 686 family-session audiotapes (randomly selected), process adherence was assessed across 10 dimensions such as attentive listening; showing encouragement, respect, and concern; asking question to engage parent; permitting parents to express thoughts without interruption; appears friendly and engaging; presents material in clear manner; appears organized and well-prepared for session; and refrains from action until sufficient information gained while also avoiding unsolicited advice-giving. Content adherence was assessed for the prescribed topics within program modules and for completion of specific items within each prescribed topic. For PCS, all sessions were videotaped for supervision and fidelity monitoring, with fidelity assessment conducted on 30% randomly sampled across mentors, cohorts, time periods, and schools. Adherence was evaluated on dimensions of process fidelity like the RMP ones, and on 21 dimensions of content fidelity pertaining to delivery of specific PCS tasks (e.g., announcements to group, required activities, communication-skill probes, session/time management), giving probe feedback appropriately, rotating opportunities for all children in the group, administering social and group reinforcers, conducting PCS activities in the correct order, and handling transitions appropriately.

### Control Condition

The six schools in the control condition participated in a universal prevention strategy focused on school-wide conflict management. This control intervention was based on adaptation of the “Conflict Manager Program” developed in the early 1980s by the San Francisco Community Board Program for, and in collaboration with, ethnically diverse elementary schools, which showed promise to improve school climate (Burrell et al., 2003; Community Board, 1995; Johnson & Johnson, 1999). The main thrust of this program was the deployment of trained fourth and fifth graders primarily on the playground to serve as mediators when children of any age engaged in conflict and voluntarily welcomed mediation. This universal prevention approach was chosen as the control intervention because: (1) no children were singled out for intervention, which avoids the risk of stigmatization; (2) by involving the whole school and not just targeted grade levels, the intervention presented the possibility of changing the overall school climate; (3) modeling of nonaggressive conflict resolution by older children potentially might have positive impact on younger children to offset possible adverse effects of antisocial behavior by older peers; and (4) children were exposed to, and participated in, prosocial conflict resolution in multiple settings (classroom, playground, lunchroom, hallways). The program generally emphasized prosocial coping with conflict situations (i.e., via peaceful communication and problem solving), with the aim to reduce schoolwide aggression, discipline incidents, and unresolved disputes through peer-mediated conflict management. With support from the project team, all six schools deployed the Conflict Manager Program and sustained it through the entire period while any of the children were still in first or second grade.

### Assessment Measures

#### Academic Performance

Report cards for third grade were obtained and evaluated to produce summary scores much like a grade point average, on a five-point scale where *A* = 4.0 and *F* = 0.0, with “minuses” and “pluses” were factored into the scoring. For each student, all report cards issued throughout the academic year (on either four or six occasions) were included. The report cards were obtained with parental consent from school records. The third-grade teachers who filled out the report cards were not involved in any aspect of the MPI, and because the records were obtained retrospectively were not aware that the grades would be examined in the study. Two summary scores were derived: (1) all academic subjects, which typically included art, language arts, mathematics, music, (i.e., reading, writing, spelling, oral expression), and social studies, but excluded physical education as well as any subject that was graded pass/fail instead of with a letter grade and (2) language arts (i.e., reading, writing, spelling, oral expression). The review of report cards and tabulating of grades was conducted by research assistants who were blind to each child’s intervention condition. Tabulation for every report card was verified by two research assistants, with discrepancies resolved by a research supervisor.

#### Behavioral and Social Adjustment

Behavioral adjustment was assessed using the teacher and parent report forms of the externalizing scale of the CBCL/CBCL-TRF (Achenbach, 1999; Achenbach & Rescorla, 2001). The externalizing scale has shown internal consistency of 0.90 for the parent CBCL and 0.94 for the teacher CBCL-TRF. Teacher-reported social competence was assessed using the social competence scale of the Walker-McConnell Scale of Social Competence and School Adjustment, which has shown internal consistency of 0.92 (Walker & McConnell, 1995; Walker et al., 1991). Parent-reported social competence was assessed using a 10-item social competence instrument developed for the study. The items, which were answered on a three-point response scale (0 = not true, 1 = somewhat or sometimes true, and 2 = very or often true), tapped aspects of communication and social relations (e.g., “Let’s others speak without interrupting them,” “Stands up for own point of view without being a bully,” and “Uses questions to understand people and things”). Internal consistency for parent-reported social competence was 0.791. The intercorrelation between the parent and teacher social competence variables was 0.25.

### Data Analytic Plan

The same data analytic method was applied separately to the boys’ and girls’ subsamples because (a) the two subsamples were independently formed; (b) collectively, the boys were likely at greater risk than the girls for adverse outcomes, and therefore needed to be evaluated separately a priori; and (c) the gender normed nature for some of the outcome measures (i.e., the externalizing scales) would have made it more difficult to interpret data patterns in a gender-heterogeneous sample. Accordingly using intent-to-treat analyses, multilevel linear mixed models were applied to all outcomes, with school treated as a random effect, children nested within schools, and the intervention variable represented as multicomponent intervention versus control. Maximum likelihood procedures were incorporated to address missingness at random. For the behavioral and social adjustment outcomes, there were two timepoints: pre-intervention in the Spring of kindergarten and post-intervention in the Spring of third grade. The academic outcome, represented by overall grades and language-arts/reading grades, was defined as the third-grade timepoint after completion of all intervention.

## Results

### Initial Demographic Characteristics of the Groups

For both the male and female subsamples, the intervention and control groups reflected comparable demographic characteristics with respect to child race/ethnicity, parent education, household income, and parent’s marital status. Supplemental Table 1 summarizes the demographic data for both subsamples presented separately by intervention condition/group. For the male subsample, the groups were comparable with respect to teacher-reported externalizing problems in kindergarten (MPI mean = 66.6, sd = 9.0; control mean = 66.0, sd = 8.7); however, for the female subsample, the groups were not comparable with respect to teacher reported externalizing problems in kindergarten (MPI mean = 65.7, sd = 8.0; Control mean = 68.8, sd = 8.6) externalizing problems.

### Fidelity of Intervention Delivery for the Targeted Components

For the RMP, process fidelity averaged 91.2% acceptability across dimensions, while content protocol adherence averaged 84.0% across key elements. For the FIP, fidelity assessments yielded 98.7% for process adherence and 96.7% and 81.7% completion of prescribed topics and specific within-topic items for content fidelity respectively. For PCS, adherence for process fidelity averaged 97.6% across dimensions and 90.0% for content fidelity dimensions.

### Analysis of the Male Subsample

#### Analysis of Academic Outcomes

For third-grade academic performance, MPI boys demonstrated better achievement than control boys with respect to overall grades, *b* = 0.2421 (*95CI* 0.022, 0.462) and language-arts/reading grades, *b* = 0.380 (*95CI* 0.113, 0.646).

#### Analysis of Behavioral/Social Adjustment Outcomes

MPI boys did not show greater gains compared with control boys from kindergarten to third grade with respect to the following: teacher-reported externalizing scale, *b* =  − 0.857 (*95CI* − 4.544, 2.833); parent-reported externalizing scale, *b* = 0.818 (*95CI* − 1.938, 3.569); teacher reported social competence, *b* = 1.854 (*95CI* 5.384, − 1.726); and parent-reported social competence, *b* = 0.099 (*95CI* − 0.027, 0.224).

### Analysis of the Female Subsample

#### Analysis of Academic Outcomes

For third-grade academic performance, MPI girls did not demonstrate better achievement than the Control girls with respect to overall grades, *b* = 0.0243 (*95CI* − 0.327, 0.369) or language-arts/reading grades, *b* =  − 0.0241 (*95CI* − 0.450, 0.394).

#### Analysis of Behavioral/Social Adjustment Outcomes

MPI girls did not show greater gains compared with Control girls from kindergarten to third grade with respect to: teacher-reported externalizing scale, *b* =  − 3.919 (*95CI* − 7.746, − 0.143), parent-reported externalizing scale, *b* =  − 2.694 (*95CI* − 5.485, 0.091), teacher reported social competence, *b* =  − 0.398 (*95CI* − 3.305, 4.094), and parent-reported social competence, *b* =  − 0.079 (*95CI* − 0.224, 0.066).

Adjusted means and standard errors for all outcome analyses are found in Table 2, online.

## Discussion

In this study, the MPI implemented in early elementary school with children at elevated risk for early onset conduct disorder involved four well-defined intervention components: reading-mentoring, family and parenting support, peer coping skills, and classroom programming. Separate subsamples of boys and girls, created through a multiple-gating procedure with teachers and parents, consisted primarily of Black children, with 63% of the families having an annual household income less than $15,000. Within both subsamples, intervention and control groups were comparable with respect to initial familial demographic characteristics. The study maintained a high participant retention rate from kindergarten through the end of third grade (92.97%). Careful assessments provided substantial evidence confirming that the intervention components were delivered with high integrity and fidelity as intended with respect to both process and content.

The main outcome that emerged in the analysis pertained to academic performance for the boys. Report card data for all of third grade, recorded by teachers with no involvement in the intervention, provided substantial and independent evidence that boys receiving the MPI performed better in overall academic areas, and more importantly in language arts including reading, compared with controls. This finding demonstrates the value of a multicontextual strategy to offset risk in this population. This outcome, occurring at the pivotal point of third grade, is notable in part because academic struggles often increase over time in boys with elevated risk. The finding is especially noteworthy in that promoting social and communication skills across family and school contexts, coupled with afterschool tutoring by mentors focused on literacy skills, was beneficial for the participating Black boys who otherwise would have been at increased risk for school failure and exclusionary practices.

For girls, the MPI did not produce better academic performance compared with controls. However, it is important to recognize that mean overall and language arts academic performance for the girls (MPI and control groups) was notably higher than that for the boys, underscoring the premise that the boys are at greater academic risk than the girls. Relatedly, there might have been more “room” to produce an academic effect with the boys.

The study did not yield intervention effects on the behavioral and social adjustment measures, for either boys or girls. For externalizing problems, children in both the MPI and control conditions showed a general decrease from kindergarten to third grade based on teacher and parent reports. If the MPI had a substantial impact on behavior problems, it was not detected with the tested measures. The teacher and parent reports of social competence did not show much in the way of any change over time, not to mention differential change.

It is worthwhile to consider how facets of the MPI might have combined to produce academic gains in the male subsample. Obviously, the RMP worked directly on reading development, but given the behavioral and classroom challenges faced by the children, it would unlikely be sufficient. The classroom and FIP components together supported the Good News Note system, which provided focused and repeated motivation to spur children’s efforts and positive behaviors—by teachers and parents jointly reinforcing specific academic and behavioral performance. The PCS promoted the acquisition of child communication and interpersonal skills, which had the potential to positively impact children’s success in the classroom. The academic achievement findings suggest that communication, language, and reading skills, supported by teacher, parent/caregiver, and tutor/mentor contributions, point to the viability of a model that emphasizes coping-competence and prosocial communications (Bierman et al., 2010; Blechman et al., 1995; Prinz et al., 2000).

The closest comparison to the present study, in terms of a multicomponent trial launched in first grade for the prevention of early onset conduct disorder, is Fast Track (Bierman et al., 2020). (As noted earlier, Dr. McMahon is one of the lead investigators of the Fast Track project.) The Fast Track multicomponent intervention included academic tutoring, group parenting program plus home visiting, child social skills training, and classroom programing. At the end of third grade after three years of intervention, Fast Track yielded effects on conduct problems and social-cognitive functioning, but like the present study found no intervention effect on teacher-reported CBCL-TRF externalizing problems (Bierman & CPPRG, 2002). Unlike the present study, however, Fast Track found no intervention effects on language arts or other academic grades by the end of third grade.

The pattern of findings and potential explanations warrant discussion. The comparison group was an active control condition, namely a school-wide conflict management program. Even though children in the control group did not receive individual programming, it is possible that the climate in their schools might have favorably changed in a way that improved mean behavioral and social functioning for the entire student body. Another possibility is that the social and behavioral measures were not sufficiently sensitive to change. It is also possible that the MPI was not sufficiently cogent to produce improvement on the social-behavioral variables, although both the fidelity evidence and the duration of intervention exposure make that explanation less viable. Finally, it is possible that despite the multifaceted, intensive, and extensive nature of the MPI, broader contextual factors such as poverty, racial discrimination, and school system factors likely present countervailing influence. On the other hand, to attain clear gains on academic grades in elementary school with boys, despite the broader adverse factors, is nonetheless a remarkable outcome.

## Limitations

Beyond strengths, limitations of the study have been identified. For behavioral and social-competence function, outcome measurement relied on teacher and parent questionnaires. Systematic observation (e.g., classroom and playground), albeit potentially burdensome and costly, would have provided additional depth to the assessment. Related to the questionnaire measures, it is possible that the baseline responses generated by kindergarten teachers as well as parents might have tempered, or perhaps injected variability, into the assessments, making it more difficult to detect subsequent improvements. Another limitation stems from when the study was conducted. In the two decades that have passed, preventive intervention strategies have evolved (Greenberg, 2010; Sprague & Walker, 2021), impediments to school-based programming and trials have surfaced (Brown et al., 2022; Combs et al., 2022), and pervasive community-wide needs and factors have been identified (Biglan et al., 2023; Boyd et al., 2023). The analytical procedures for cluster-randomized prevention studies have also substantially evolved, which could have impacted the present study with respect to design planning and implementation of (Murray et al., 2020; Raudenbush & Bloom, 2015). A final limitation pertains to the variability within the sample that might have obscured effects. There were likely some children who benefitted more than others from the MPI. Planned exploratory analyses will generate hypotheses about variation in impact and permit better understanding about the factors that were potentially pivotal.

## Conclusions

This study demonstrated that the academic achievement of young boys can be strengthened in the face of contextual adversity, drawing on a concerted and coordinated multicomponent prevention strategy. The continuing need to cogently support Black male youth development is unquestionable (Gaylord-Harden et al., 2018) but should not be taken to suggest that young Black females do not face challenges in need of mitigation.

With respect to young children who are at clear risk for both academic and behavioral difficulties, there is no substitute for dedicated, caring, and competent individuals (e.g., mentors as reading tutors, teachers, family support professionals) devoting many hours of targeted assistance to move the needle. The need for such concerted effort has not diminished over the decades. That said, several changes in society present challenges for prevention scientists: (a) technology-based delivery (both asynchronous and virtual) is replacing traditional in-person and in-home delivery; (b) parents, children, and perhaps teachers may reflect impatience and a lower willingness to persist with longer interventions; (c) schools have become more physically insular in response to the high incidence of mass shootings and other violence; (d) political, administrative, and social-media forces have adversely affecting the ability and inclination of schools to collaborate; and (e) volunteerism among high school and college students, which has gone through ups and downs from one generation to the next, is difficult to gauge and count on.

There are advantages and disadvantages to an intervention model like the MPI conducted for this study. The MPI uniformly invoked principles and programming practices designed to nurture children and families, to recognize and accelerate positive behaviors in children who face failure and criticism, and to promote effective communication and prosocial interactions. Another advantage is comprehensiveness achieved by addressing reading, classroom motivation and behavior, social interactions, communication skills, and family support at the same time. On the other hand, the initiation and coordination of four components through schools might be too big of a hill to climb these days. Another potential disadvantage is that the MPI adopted more intensive programming across the entire sample without tailoring “dosage” to match individual differences in needs. For example, the family component provided two years of home-based family intervention for all children. A more practical and efficient approach would be to enact a public health friendlier parenting support strategy that permits parents to access whatever level/intensity of services they might need (Prinz, 2012).

The nature and diligence of the prevention strategies reported in this article are quite compatible with the spirit and quality of the research that Dr. McMahon has undertaken throughout his career. Consistent with the tenets reflected in much of his work (e.g., McMahon & Kotler, 2008; McMahon et al., 1999, 2021; Peters & McMahon, 1996), the present study underscored: (a) the importance of intervening promptly at the beginning of first grade (or earlier if possible); (b) the value of engagement of parents through home-school communications and direct parenting support; (c) the need for focused attention to reading and other academic skills and not just behavioral difficulties; (d) the roles that nurturing individuals can and should play in efficacious prevention of early onset conduct disorder (Smith & Bradshaw, 2017); and (e) that there are no shortcuts for careful attention to rigor and delivery of preventive interventions when it comes to children experiencing elevated risk.

## Supplementary Information

Below is the link to the electronic supplementary material.

Supplementary file1 (PDF 154 KB)
