# Supplementary material for: A Multicomponent Preventive Intervention in the Early Elementary Years: A Look at Academic and Social Adjustment Outcomes
Source: Prev Sci. 2024 Nov 11;26(4):633–43. doi: 10.1007/s11121-024-01748-w (PMC12209043; doi:10.1007/s11121-024-01748-w)
Supplement: Supplementary file 1 — Supplementary file1 (PDF 154 KB) [file 11121_2024_1748_MOESM1_ESM.pdf]

Figure 1. CONSORT Flowchart for Multi-contextual Preventive Intervention Trial

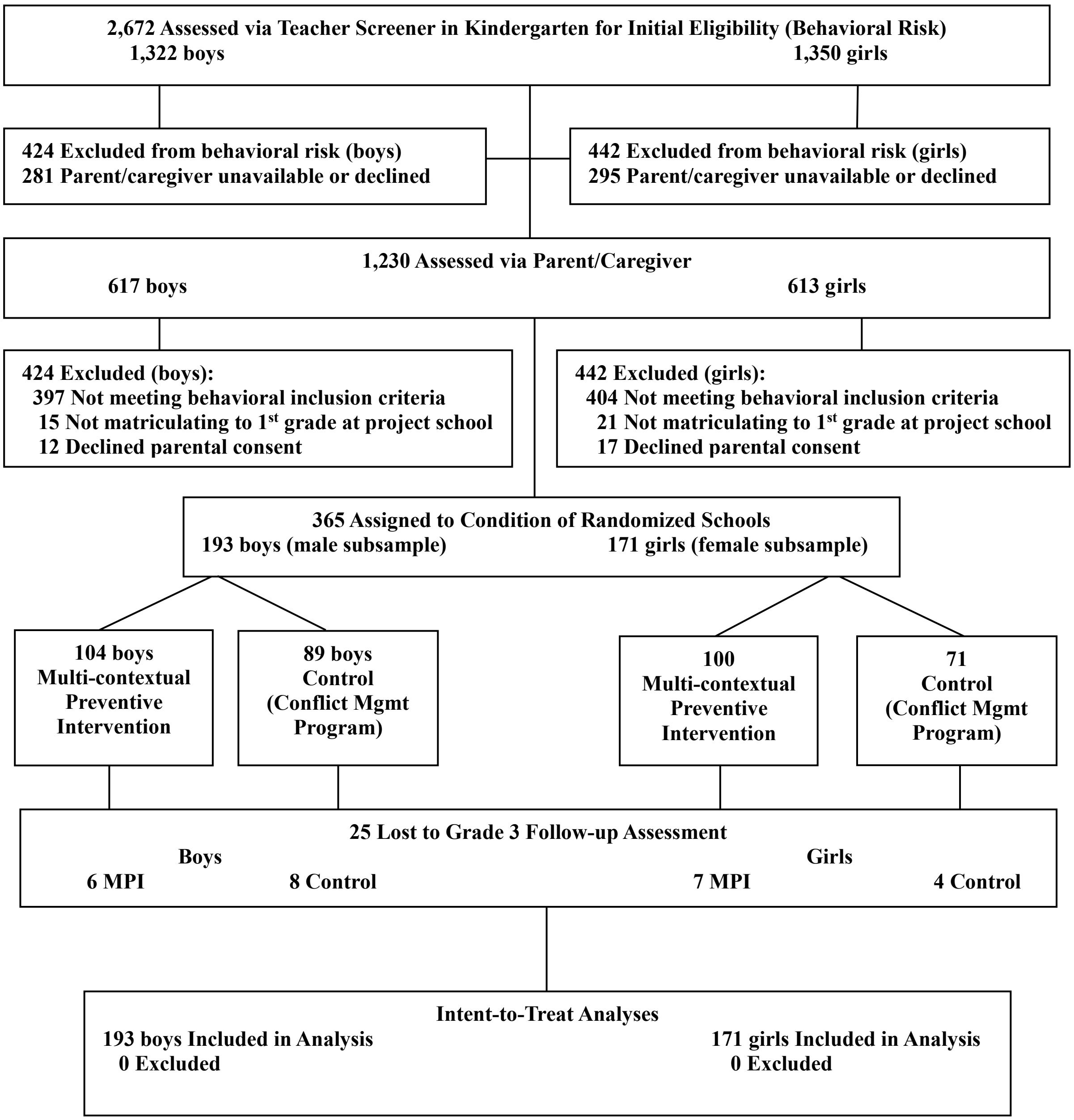

]

Table 1 *Initial Demographic Characteristics for the Multi-Contextual Preventive Intervention and Control Groups in the Male and Female Subsamples*

		**MPI Group**	**Control Group**
**MALE SUBSAMPLE**			
Number of Boys in Each Group/Condition		104	89
Child Race	Black non-Hispanic	95.3%	95.6%
	White non-Hispanic	2.9%	2.2%
	White, Hispanic	0.9%	1.1%
	Native American	0%	0%
	Other category	0.9%	1.1%
Parent Education	< HS graduation	22.2%	16.9%
	HS graduate	47.1%	41.6%
	Some college	26.9%	32.5%
	College graduate	3.8%	9.0%
Household Income	< $15,000	64.7%	60.2%
	$15,000 - $29,999	23.6%	27.3%
	$30,000 - $49,999	8.8%	10.2%
	$50,000 +	2.9%	2.3%
Marital Status	Married	26.9%	29.2%
	Divorced	12.5%	11.2%
	Widowed	1.0%	6.7%
	Separated	10.6%	10.1%
	Never married	49.0%	43.8%
**FEMALE SUBSAMPLE**			
Number of Girls in Each Group/Condition		100	71
Child Race	Black non-Hispanic	93.0%	95.8%
	White non-Hispanic	6.0%	1.4%
	White, Hispanic	0%	1.4%
	Native American	0%	1.4%
	Other category	1.0%	0.0%
Parent Education	< HS graduation	23.0%	12.7%
	HS graduate	40.0%	36.6%
	Some college	30.0%	39.4%
	College graduate	7.0%	11.3%
Household Income	< $15,000	63.5%	55.7%
	$15,000 - $29,999	25.0%	27.1%
	$30,000 - $49,999	8.4%	12.9%
	$50,000 +	3.1%	4.3%
Marital Status	Married	31.0%	31.0%
	Divorced	11.0%	8.5%
	Widowed	3.0%	1.4%
	Separated	17.0%	21.1%
	Never married	38.0%	38.0%

Table 2 *Adjusted Means and Standard Errors for Academic Performance and Behavioral/Social Outcomes as a Function of Multicomponent Preventive Intervention versus Control, for Boys (Male Subsample) and Girls (Female Subsample)*

		**Intervention Condition**	
**Outcome**	**Time Point**	**Multicomponent Intervention Adj Mean (SE)**	**Control Adj Mean (SE)**
**MALE SUBSAMPLE**			
Report card grades: all academic subjects	Third Grade	2.35 (0.10)	2.11 (0.10)
Report card grades: language-arts, reading	Third Grade	2.38 (0.12)	2.00 (0.11)
Teacher-Reported Externalizing Scale	Kindergarten<br>Third Grade	66.1 (1.12)<br>64.5 (1.22)	65.6 (1.15)<br>63.0 (1.27)
Parent-Reported Externalizing Scale	Kindergarten<br>Third Grade	63.5 (1.04)<br>58.1 (1.11)	61.3 (1.05)<br>56.8 (1.10)
Teacher-Reported Social Competence	Kindergarten<br>Third Grade	35.9 (1.01)<br>37.5 (1.10)	36.6 (1.04)<br>36.4 (1.15)
Parent-Reported Social Competence	Kindergarten<br>Third Grade	1.88 (0.049)<br>1.83 (0.052)	1.84 (0.050)<br>1.89 (0.052)
**FEMALE SUBSAMPLE**			
Report card grades: all academic subjects	Third Grade	2.57 (0.13)	2.55 (0.14)
Report card grades: language-arts, reading	Third Grade	2.48 (0.16)	2.50 (0.17)
Teacher-Reported Externalizing Scale	Kindergarten<br>Third Grade	65.6 (1.05)<br>64.0 (1.10)	68.9 (1.22)<br>63.1 (1.33)
Parent-Reported Externalizing Scale	Kindergarten<br>Third Grade	63.2 (1.00)<br>58.8 (1.03)	64.4 (1.16)<br>57.2 (1.20)
Teacher-Reported Social Competence	Kindergarten<br>Third Grade	39.5 (0.93)<br>39.9 (0.99)	39.9 (1.08)<br>40.8 (1.17)
Parent-Reported Social Competence	Kindergarten<br>Third Grade	1.99 (0.047)<br>1.99 (0.048)	1.99 (0.054)<br>1.91 (0.057)
